# The Escape

**Published:** 1919-08-09

**Authors:** 


### THE ESCAPE.

Now that the fog of war has been dispersed and the Press Censor demobbed, and, moreover, now that the research people have again taken over No. 5 War Hospital at Highgate, I see no harm in telling a secret once far more closely kept than all that stuff about tanks and submarines—the secret of the fire-escape which led past No. 15 ward of the said No. 5 War Hospital, now defunct.

As everybody knows who has had the good fortune to be a patient there, the buildings are surrounded by a high cleft-oak fence with tenderhooks bristling along its crest. Only one gate is left open at night, the one which faces the front door of the main building. An excellent light over the portal greets the tired soldier on his return at eve (air-raids barred), and an orderly, with pencil and grubby notebook ever ready, sits just inside the door waiting for unwary warriors who arrive home after 10 P.M. Several wights have tried at various times to tamper with that closing hour, but until "Daddy" Coles made his famous bet in Ward 15 none had met with any measure of success.

It happened thus: Several warriors, weary of toasting their different kinds of stumps and talking wound gratuities around the fire in Ward 15, essayed athletics. "Nobby" Cooke kicked over a matchbox stood upright on the floor three feet from him (and fell over back wards and reopened his scalp wound); young Grayson undertook to recite the first verse of "Casabianca" with a billiard ball in his mouth (the surgeon on duty nearly had to anæsthetise him to get it out again); and Vernon tried to stand on his hands on two lockers (and came a spinning nose-dive which broke them both). Everybody contributed to the entertainment except "Daddy" Coles, a decrepit old broken soldier of about twenty-eight, and when they jeered at him he said he scorned mere stunts. What he wanted to do was to make the world a brighter and better place, and when they jeered again he pointed to the fire-escape and said, "What about that, then?"

Most of the others, nursing new bruises or ordered back to bed, didn't see the point; but Irvine, who was late the night before and had just had a prosaic interview with the adjutant, woke to interest at once.

"Can't be done," he said.

"Can," said "Daddy" Coles briefly. "Bet you ten bob I do it—old age, crutch and all."

They made the bet, and in the end he did it. He leaned out of the window till he got to the danger angle, made a snatch with his crutch at the hand-rail, caught it, and then handed himself up the crutch and swung himself across the escape.

Getting back was easy, the window-sill being broad and flat; and, except for Irvine, nobody took any further interest in the feat.

But Irvine did it with a crook-handled walking-stick instead of a crutch—did it twice. "It's dead easy," he said in surprise.

"You've got two legs, and your left arm's as good as new," "Daddy" objected.

Irvine went back to the fireside and pondered deeply, and, in spite of being confined to hospital for three days—a result of the interview with the adjutant—he seemed strangely unconcerned about it.

When the three days expired and he was poshing himself up to visit the gay metropolis once more, he called me aside.

"See here," said he. "I may be late to-night. I'm coming home by a new route. You sleep next the window. If you should hear a tapping on the pane after lights out—but I see by your bright look of anticipation that I need say no more."

He did it successfully. In the following week he was out four times till after midnight, and the week after that every night. The following week, having been more than a fortnight without an interview with the adjutant, he asked and got forty-eight hours' leave, ostensibly to visit a rich and elderly aunt at Croydon. He left hospital at noon, and at two o'clock exactly two elderly gentlemen in blue overalls, loaded with pots and kettles, climbed the fire-escape and set to paint it a gaudy red. By nightfall they had got down to the top of the window of Ward 15. By the evening of the next day they were out of sight below, and the view from the window had a glow as of summer sunshine, the winter landscape being seen through a network of fire-hued iron bars.

Excitement rose as eventide drew on. Ten came, ten-fifteen, and lights out, and still no Irvine appeared. The hospital subsided into silence, eleven struck from neighbouring steeples, and still no Irvine. . . .

It was young Vernon who thought of pulling down his bedclothes and punching his pillows into disarray, but he had been back in his own bed a full half-hour before we heard feet upon the fire-escape. And at that very moment the door swung open and the night sister entered.

Five simultaneous gasps from five beds ended in fairly natural snores. Fortunately her electric torch flashed on the window-blind, and the feet outside were still at once. She went up the ward, flashing her torch upon one or two assiduous sleepers, and stopped for a moment at Irvine's bed, but, apparently reassured by the tumbled bedclothes, left the ward without remark.

Giving her about thirty seconds' law, I reached for the blind and shook it, and a hoarse whisper from outside asked if it was all right.

"Don't make a row. Sister's only this minute gone."

He came in quietly as an otter slipping into a pool, and all would have been well if only someone had thought of removing the sterilising drum from under the window. As it was, he put a foot on it and knocked it over with a crash.

"That's torn it!" moaned a voice so charged with emotion as to be unrecognisable. It was drowned in a renewed chorus of snoring as five craven souls got ready to convince the imminent sister of their non-complicity in crime. But you learn to act quickly in the Army. In three strides Irvine was by his bed; a jerk, and his cap was under it; a dive, and he was in it—boots, clothes, Sam Browne and all—and, with the sheet drawn to his ears, his snores were mingling with the others.

Sister didn't return. One by one the sham snores gave way to real ones, and only when bed-making began in the morning did we remember we had overlooked a matter more important than the tin cylinder beneath the window.

Sister Williams' first words roused all the ward like a trumpet-call.

"Has your wound broken out again, Captain Irvine?"

"No, Sister. My nose came on to bleed a little in the night."

"It must have been more than a little," cooed the soft voice, unrelentingly.

The sheets were streaked and barred with gory red. His coat was ruined; his breeches blotched incarnadine; his posh field-boots striped like red and brown zebra hide. . . .

But he had plenty of time to get it off. The Adjutant saw to that.

